# Biosorption of *Escherichia coli* Using ZnO-Trimethyl Chitosan Nanocomposite Hydrogel Formed by the Green Synthesis Route

**DOI:** 10.3390/gels9070581

**Published:** 2023-07-17

**Authors:** Ibrahim Birma Bwatanglang, Faruq Mohammad, John Nahadi Janet, Wasmia Mohammed Dahan, Hamad A. Al-Lohedan, Ahmed A. Soleiman

**Affiliations:** 1 Department of Pure and Applied Chemistry, Adamawa State University, Mubi 650001, Nigeria; 2 Department of Chemistry, College of Science, King Saud University, Riyadh 11451, Saudi Arabia; 3 College of Sciences and Engineering, Southern University, Baton Rouge, LA 70813, USA

**Keywords:** *Terminalia mantaly*, zinc oxide, trimethyl chitosan, *Escherichia coli*, biosorption, Langmuir isotherm

## Abstract

In this study, we tested the biosorption capacity of trimethyl chitosan (TMC)-ZnO nanocomposite (NC) for the adsorptive removal of *Escherichia coli* (*E. coli*) in aqueous suspension. For the formation of ZnO NPs, we followed the green synthesis route involving *Terminalia mantaly* (TM) aqueous leaf extract as a reducing agent, and the formed ZnO particles were surface-coated with TMC biopolymer. On testing of the physicochemical characteristics, the TM@ZnO/TMC (NC) hydrogel showed a random spherical morphology with an average size of 31.8 ± 2.6 nm and a crystal size of 28.0 ± 7.7 nm. The zeta potential of the composite was measured to be 23.5 mV with a BET surface area of 3.01 m^2^ g^−1^. The spectral profiles of TM@ZnO/TMC NC hydrogel on interaction with *Escherichia coli* (*E. coli*) revealed some conformational changes to the functional groups assigned to the stretching vibrations of N-H, C-O-C, C-O ring, and C=O bonds. The adsorption kinetics of TM@ZnO/TMC NC hydrogel revealed the pseudo-second-order as the best fit mechanism for the *E. coli* biosorption. The surface homogeneity and monolayer adsorption of the TM@ZnO/TMC NC hydrogel reflects majorly the entire adsorption mechanism, observed to display the highest correlation for Jovanovic, Redlich–Peterson, and Langmuir’s isotherm models. Further, with the use of TM@ZnO/TMC NC hydrogel, we measured the highest adsorption capacity of *E. coli* to be 4.90 × 10 mg g^−1^, where an in-depth mechanistic pathway was proposed by making use of the FTIR analysis.

## 1. Introduction

In recent years, there has been an increased usage of metal or metal oxide nanoparticles (NPs) towards biomedical applications, as they are all well capable of offering antimicrobial properties which initiates primarily through the surface contact/adhesion. This contact of metal NPs such as silver (Ag), gold (Au), zinc (Zn), copper (Cu), titanium (Ti), manganese (Mn), cobalt (Co), etc., with that of biological cells causes a series of intracellular pathways such as oxidative stress, metabolic changes, protein fragmentations, etc. The biomolecules such as lipopolysaccharide, lipoteichoic acid, protein, and phospholipid bilayers on the bacteria cells were reported to dominate the NP-cell adhesion mechanism by either through their highly charged structure and/or through the bridging effect of their surface functional groups [1,2]. This mechanism was reported to govern the NPs–bacterial interactions in studies investigating the antimicrobial susceptibility, adhesion, and adsorption behavior towards the metal oxide NPs surface [1]. Among the various kinds of metal NPs, Zn and its oxide stand out as one of the most potent microbial killers, demonstrated by either through the release of Zn^2+^ ions from the dissolution of ZnO or by the induction of reactive oxygen species (ROS) [3,4]. Biomolecules in the peptidoglycan layer and in the cell membrane of bacteria such as teichoic acid, lipoteichoic acid acting as the chelating agents preferentially get attracted to the Zn^2+^ ions by electrostatic interactions [3,5]. Such bacterial action of ZnO NPs occurs through a series of steps such as the adsorption at bacterial surface, the occurrence of electrostatic interactions between the cell surface proteins and the positive sites of ZnO [3].

In the pursuit to fully utilize the biological properties of ZnO NPs, several techniques are being developed to generate the particles with change of size, shape, architecture, and surface nature. This includes the physical and chemical approaches such as the spray pyrolysis, sol–gel, zinc–alcohol reaction, microwave-assisted procedures, hydrothermal, ultrasonic conditions, chemical vapor deposition, and precipitation techniques. However, beside the issues of high cost, labor-intensive processes, high energy consumption rate, and lengthy process time, issues relating to the use of chemical agents for reduction and precipitation reactions in the operations leave behind hazardous footprints [6]. Though the surface coatings with the incorporation of surfactants and other hydrophilic/hydrophobic moieties are reported to have a strong effect towards the controlling of ZnO NPs toxicity [7], studies show that some of these coating agents used are by themselves risk promoters and often involved the use of varied synthetic chemicals in the synthesis procedures [8].

In consequence to these drawbacks, pertinent research efforts have over the years focused on compatible, eco-friendly, and speedy synthesis techniques requiring the use of biological active ingredients derived from plants, yeast, fungi, bacteria, and seaweeds as reducing agents for the synthesis of metal oxide NPs [9]. However, the use of yeast, fungi, and bacteria entails some stringent procedures, often involving the need for expensive and time-consuming culture media, besides being nonpractical for a large-scale production [10]. The plant-mediated synthesis, on the other hand, is found to be more practical and involves cheap protocols that can be diversified for a large-scale production. The reducing agents in these methods are the plant metabolites such as alkaloids, polysaccharides, amino acids, vitamins, flavonoids, and terpenoids, where they are all reported to possess the inert properties to reduce metal ions/oxides to zero-valent metal NPs [11]. In addition to other plant extracts, a substantial amount of research reported ZnO NPs synthesis using extracts of various species of the genus Terminalia, which include T. catappa, T. chebula, T. arjuna, terdinandiana, and T. bellirica [12,13,14,15]. Of the various species, no available literature reported indicating the synthesis of ZnO NPs using *Terminalia mantaly* (TM) for the adsorption of bacteria. The studies available involved the synthesis of biogenic AgNPs [16] and Au NPs [17] as antimicrobial agents. For example, Lavanya et al. [18] investigated the dielectric properties of 14–18 nm ZnO NPs synthesized using TM leaf extracts, while no studies reported on the antibacterial properties of these particles nor their biosorption behavior. The TM belongs to the flowering plant family, Combretaceae, from the genus Terminalia. Several studies reported this species to possess intrinsic biological properties of varied medicinal importance [19]. The bioactive molecules are reported to constitute the phytochemicals such as tannins, pentacyclic triterpenes, glycoside derivatives, flavonoids, and phenolic compounds [16,17].

As mentioned earlier, the focus of this study was to look into the biosynthesis of ZnO NPs using the bioactive molecules of TM aqueous leaf extracts as reducing reagents to facilitate the conversion of the initiator, zinc acetate dehydrate to zinc oxide NPs [20]. To further improve upon the bacterial adsorption potential of the biogenic NPs, the ZnO NPs are surface-coated with trimethyl chitosan (TMC). Surface coating using biopolymer was reported to enhance the chemical and biological properties of metal oxide NPs. Pristine chitosan, though reported to be biodegradable, readily accessible naturally, possess low toxicity, and have chemical functionality as adsorbents, was further observed to demonstrate few drawbacks in its application as adsorbents due to its preferential solubility in acidic medium [21]. However, appropriate modification in the form of TMC-based particles was reported to demonstrate higher adsorption capacity, biocompatibility, and nontoxicity in biological milieu than pristine chitosan. The TMC-based particles possess ionic characters and solubility over a wide pH range, thereby influencing the protonation status of the cell microenvironments, and provide superior adhesion for biological entities compared to the unmodified chitosan [22]. The initiation of these ionic characters, especially under low pH, induces the formation of opposing ionic species within the bacterial cell, increasing the osmotic pressure of the endosomal membranes coursing it to burst in the process, a process referred to as the “Proton sponge” effect [23]. These observed advantages informed the choice of the modification of chitosan to TMC to investigate its potential as a biopolymer–metal-oxide-based adsorbent for the adsorption of bacteria in aqueous suspensions. Based on the observed material improvement, this study also looked at the adsorption potential of the formed ZnO/TMC NC towards *Escherichia coli* (*E. coli*) and linked the mechanism through various kinetic and adsorption models. We further investigated the ZnO-*E. coli* interactions using the Fourier transform infrared (FTIR) technique towards providing at least some basic information for the ZnO/TMC hydrogel involvement in the *E. coli* adhesion.

## 2. Results and Discussion

### 2.1. Phytochemical Analysis of TM Plant Extract

Table 1 shows the varied distribution of different secondary metabolites in the TM extract where the analysis indicates for the availability of major phytochemicals consisting of alkaloids, saponins, and phenols followed by tannins, flavonoids, and terpenoids. The least detected phytochemicals are steroids and anthraquinones. The concentration of phytochemicals from the qualitative analysis shows that phenols are the major metabolites (36.5 mg/100 g) followed by alkaloids with 8.7 mg/100 g, which therefore suggested that the bioreductions are majorly driven by the hydroxyl, aldehyde, and carboxyl functional groups present in the extracts. These metabolites possess inert properties to bioreduce metal ions or metal oxides to zero-valent metal NPs. The hydroxyl (^−^OH) group of flavonoids and the phenols were reported to possess this ability to initiate such a reaction [17,24].

### 2.2. Characterization of TM@ZnO/TMC NC Hydrogel

**Figure 1:**
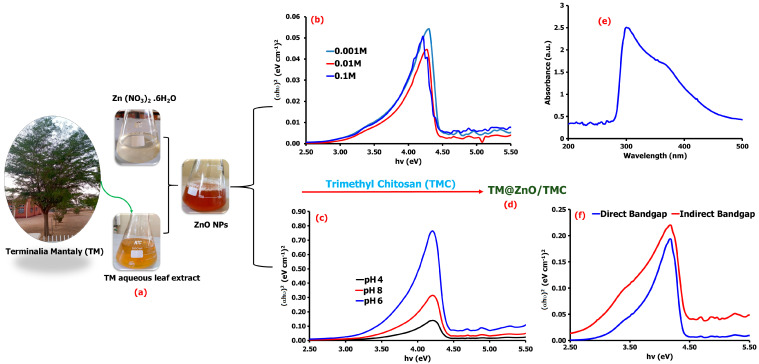
Schematic representation of the synthesis of ZnO NPs from TM leaf extract (**a**), the direct and indirect bandgap energies of the synthesized TM@ZnO NPs at varying concentrations of the precursor salts (**b**) and pH (**c**). The produced TM@ZnO/TMC NC hydrogel product (**d**), its UV–vis spectrum (**e**), and the corresponding bandgap spectra (**f**).

UV–vis spectroscopy was used to investigate the effect of varying pH and precursor concentration on the bioreduction processes of TM on ZnO NPs. The schematic in Figure 1 shows the colorless solution of the Zn(NO_3_)_2_⋅6H_2_O changing to a red-wine color on addition of the amber-colored TM aqueous extract. The appearance of red-wine precipitate confirms the formation of ZnO NPs. As indicated earlier, the phytochemicals identified in the extracts of TM perform dual functions, i.e., as reducing and stabilizing agent, and thus facilitate the reduction process of converting Zn^2+^ to the Zn^0^ [25]. Upon constant stirring at 60 °C, the metallic zinc becomes oxidized to ZnO, and the additional calcination process at 350 °C for 2 h reduces the ZnO to ZnO NPs. Also, flavonoids, terpenes, and tannins possess the inert characteristics to interact with the -OH groups that surround Zn^2+^ by losing electrons to help the oxidation of -OH ions into carbonyl groups, and they are hence made available for the NPs stabilization [26]. In solution, Zn^2+^ ions are hydrolyzed (Zn^2+^ + 2OH), forming charges that bind to the TMC through its amino or the hydroxyl groups to form TM@ZnO/TMC NC hydrogel [27,28]. The formation of ZnO NPs was further confirmed by the UV–vis absorption and changes in the bandgap energy of formed ZnO NPs. (Figure 1b). The bandgap energy estimated using Tauc methods extracted from the absorption spectra changes on varying the concentration of Zn precursor salt, which implies that the larger-sized particles can be produced as the bandgap increases. The bandgaps of materials were reported to be directly influenced by the total concentration of ions, such as H^+^ and OH^−^, establishing an intimate relationship between the counter ions of the first solvation shell with electrons in the charged conducting material. The changes in electron density generated by the ion’s approximation process define the material’s bandgap [29].

Further assessment of the UV–vis spectrum at lower pH displayed a bandgap energy of 3.97 eV at pH 4 and 3.65 eV at pH 6 compared to the relatively higher bandgap of 3.97 eV recorded at pH 8 (Figure 1c). The results aggress with submissions by Fuku et al. [30], reporting a bandgap of 0–3 eV. Several studies reported the variable bandgap energy of ZnO NPs synthesized through green chemistry. Portulaca oleracea leaf extract-mediated ZnO NPs were reported by Gherbi et al. [29] to display an indirect bandgap of 2.15–2.74 eV and direct bandgap of 2.97–3.91 eV. Similarly, other literature studies reported a bandgap of 3.21 eV from *Hagenia abyssinica* leaf extracts [31], 3.8 eV from *Hibiscus sabdariffa* flower extract [32], and 3.3 eV from *Cnidoscolus aconitifolius* aqueous leaf extracts [33]. Such differences of bandgap variations could emanate from several factors, ranging from the material particle size and surface roughness to the effect of lattice strain among the ZnO NPs formed from different plant extracts [34].

The coating of TM@ZnO NPs with TMC showed a maximum absorption band of 308 nm (Figure 1e). Other studies also reported a maximum absorption band for ZnO synthesized using pumpkin seeds’ extract at 272 nm [35], a wavelength of 300 nm using aqueous leaf extracts of coffee [36], and absorption peaks at 302 for ZnO-NPs synthesized with fruit extract [10]. The TM-mediated ZnO/TMC NC hydrogel synthesis in this study displayed an indirect bandgap of 2.87 eV and direct bandgap of 3.43 eV at pH 6 and precursor salt concentration of 0.001 M (Figure 1f), a bit lower than the values reported for the uncoated ZnO NPs, which implies that the surface coating with the polymer composite (TMC) increases the degree of disorder, and hence, a reduction in the energy gap was noticed. Furthermore, the presence of charge-transfer complexes between the metal NPs and the TMC matrix also influences the bandgap energy decline and optical properties improvement [37]. For example, chitosan-modified ZnO NPs prepared through sol–gel synthesis were reported in a study that has bandgap energy in the range of 2.7 to 3.2 eV [27].

**Figure 2:**
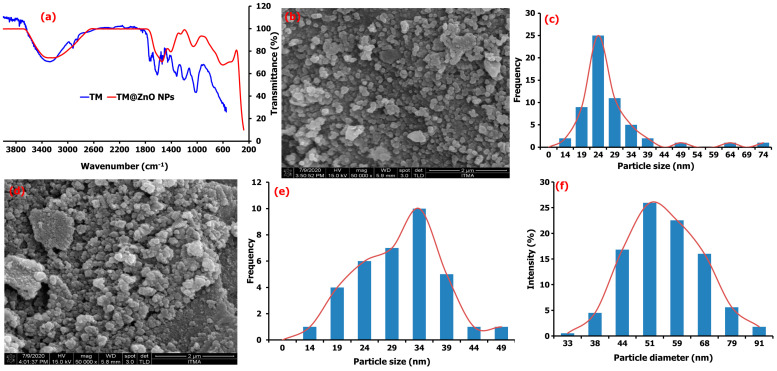
(**a**) FTIR analysis of pure TM leaf extract compared to TM@ZnO NC hydrogel, (**b**) the FESEM image and (**c**) particle size distribution of TM@ZnO NPs. (**d**) FESEM macrograph, Mag × 50,000 (**e**) particle size distribution, and (**f**) DLS particle diameter of TM@ZnO/TMC NC hydrogel.

The Fourier transform infrared (FTIR) spectroscopy gave evidence related to the activities of molecules in the TM leaf extracts added during the ZnO NPs synthesis. Further, this provides information for the formation of TM@ZnO NPs polymer composite (TM@ZnO/TMC NC hydrogel) and its subsequent role in the adsorption of *E. coli* from aqueous suspension (TM@ZnO-TMC/*E. coli*). Figure 2a provides the FTIR spectra of TM leaf extract, where it shows a hydrogen-bonded -OH stretch of alcohol and phenol at 3376–3107 cm^−1^. The existence of multiple stretching bands of secondary amine matched with aromatic–CH_3_ is found at 2923–2853 cm^−1^. The band at 1735 cm^−1^ corresponds to aryl α, β-unsaturated acids, anhydrides, and the peak at 1622 cm^−1^ represents the stretching bands of α, β-unsaturated aldehydes. The peaks at 1542 cm^−1^ and 1320 cm^−1^ define the C−N stretch of tertiary thiols and the skeletal stretching of aromatic aryl -NH_2_, respectively. The peaks at 1208 cm^−1^ and 1028 cm^−1^ represent tertiary amine and the vibration bands of C–O and C–C of the pyrinoid ring and glycoside [16,17].

Most of these functional groups found in the TM extract were not detected in the spectra of the ZnO NPs, and their absence may be due to the interaction with the metal salt/ions. The bands at 3159–3476 cm^−1^ in the spectra of TM@ZnO NPs displayed the presence of surface hydroxyl groups of alcohol, phenols, or carbohydrates. The 1556 and 1416 cm^−1^ relate to the C−H stretching and symmetric C=O stretching modes. The peak at 1262–1000 cm^−1^ matched the activities of C–O and C–C vibration bands of the pyrinoid ring and glycoside. Also, the peaks identified at 614 and 471 cm^−1^ represent the bands associated with Zn–O vibrations. Similar activities were reported for other biosynthesized ZnO NPs [25]. The mechanism involving electrons transfer from either C=O or C=C groups, N–H, O–H, C=C, and C–O to the Zn^2+^ ions’ free orbital favored the interaction processes [38]. These functional groups, C=O or C=C groups, N–H, O–H, C=C, and C–O identified in the TM extract acted as the species responsible for the bioreduction processes leading to the formation of metallic NPs. The FTIR results indicated that the –OH groups of the extract interact through electron transfer with ZnO NPs brooding the peak, as observed in the spectra of ZnO NPs.

The morphology of as-synthesized ZnO-biomediated TM shown in the field emission scanning electron microscopy (FESEM) images (Figure 2b) are quasi-spherical in shape with particle size distribution ranging from 14 to 25 nm, with a mean value of 25.5 ± 3.2 nm (Figure 2c). The formation of the quasi-spherical-shaped structures of ZnO-NPs is due to Zn^2+^ interaction with the OH^−^ ions in the plant extract and subsequent heat treatment that slowly converts the zincate ions into ZnO. The ZnO crystal structure is slowly formed by the surrounding OH^−^ ions, behaving as a polar crystal and on the saturation, allowing the ZnO nucleus to grow, and initiates particle formations [26]. The FESEM of TM@ZnO NPs-coated TMC displayed in Figure 2d showed a relatively higher agglomeration but random spherical morphology compared to the uncoated TM@ZnO NPs. The particle size due to the polymer composite formation produced an overlapping particle size in the range of 14–49 nm with a mean value of 31.81 ± 2.68 nm (Figure 1e). The increase in grain size from the overlaying of particles influenced the nucleation processes and relatively maintained the nanometric shapes formation processes [19,26]. The hydrodynamic size of TM@ZnO/TMC NC (Figure 1f) determined using dynamic light scattering (DLS) shows the particle diameter with an average size of 57.75 ± 19.18 nm for the aqueous preparation, which is a bit larger compared to the size distribution obtained from FESEM (Figure 1e). The increase was largely due to the deposition of hydrate layers on ZnO-NPs polymer surface [32].

The study by Álvarez-Chimal [26] reported a quasi-spherical FESEM topology for the ZnO NPs prepared from the extract of *Dysphania ambrosioides*. According to the study, the particle size distribution in the range of 4–140 nm was observed to be influenced by the synthesis temperature. The study by Faisal [39] reported a semispherical and agglomerated SEM image for ZnO NPs with overlapping particle size ranging from 43.3 to 83.1 nm prepared using the aqueous extracts of *Myristica fragrans* fruit. Similar spherical but agglomerated SEM images were also reported by Mthana et al. [10] and Wali [40] for ZnO NPs biomediated by *Capsicum chinense* fruit extract and *Papaver somniferum*, respectively. The mean SEM size distribution of ZnO NPs biologically synthesized as reported Gherbi et al. [29] is primarily in the range of 22.17 to 27.38 nm with slightly agglomerated topology. Also, *Aqueous Piper beetle* leaf extract was reported by Thi Tran [41] to mediate the formation of a spherical shape ZnO NPs. A spherical/cubic and hexagonal shape for the ZnO NPs prepared from the extracts of *Mentha pulegium* were reported in a study to give a particle size distribution of 320 nm on the surface coating with chitosan [21]. Similarly, ZnO NC coated with chitosan (chitosan-ZnO) was observed to show a dendritic floc-like structure in a study reported by Bashal [28]. A random spherical shape was also reported for ZnO NPs coated with chitosan derived from crab shells (30–80 nm), shrimp shells (20–70), and *Streptomyces griseus* bacteria (35–75 nm) [27]. A spherical or elliptical shape of the ZnO NPs on the surface of chitosan was also reported by Zango et al. [42], where the FESEM images show that the ZnO/chitosan NC prepared by the in situ precipitation method has a spherical shape with the average size of 20–25 nm [43]. For this, the HRSEM revealed the formation of uniformly distributed spherical- and hexagonal-shaped ZnO particles with an average size of 13–28 nm. Based on all these analyses, it is quite natural that the TM@ZnO/TMC NC hydrogel formed by the green synthesis route has a quasi-spherical shape and average particle size around 25 nm.

**Figure 3:**
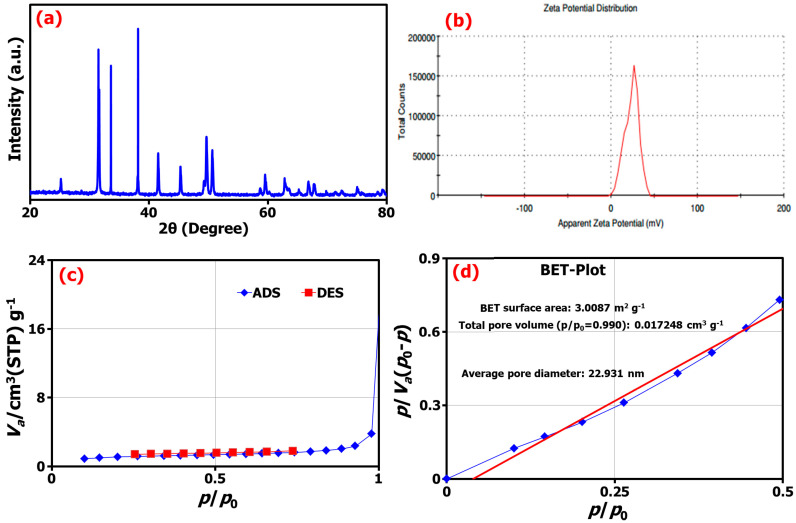
(**a**) The XRD pattern, (**b**) the zeta potential from DLS analysis, (**c**) the adsorption/desorption isotherms, and (**d**) BET analysis of TM@ZnO/TMC NC hydrogel.

The powdered X-ray diffraction (XRD) diffraction patterns for the synthesized TM@ZnO/TMC NC hydrogel showed the Bragg reflections indicated to 2θ values at 31.3°, 34.3°, 38.3°, 45.6°, and 50.1° (Figure 3a). These values exhibited some common characteristic peaks matching the hexagonal wurtzite structure of ZnO (JCPDS card No. 36-1451) indexed as (1 0 0), (0 0 2), (1 0 1), (1 0 2), and (1 1 0), respectively [25]. The narrow width and strong intensity of the diffraction peaks indicate the formation of crystalline ZnO NPs. Several studies reported similar observations for biosynthesized ZnO NPs [6,25,26,33,44]. The presence of hydrated and anhydrous structure for the TMC hydrogel was identified by the appearance of an additional peak at ∼2θ = 20°, implying the formation of a polymer-coated ZnO composite [45]. The crystallite size of TM@ZnO/TMC NC hydrogel analyzed according to the Debye–Sherrer equation by XRD analysis for the most intense peaks to give an average crystal size of 28.02 ± 7.72 nm nearly matched the particle size determined by FETEM examination (31 nm). In addition, the formation of polymer-coated ZnO NC was reported to be influenced by the properties of capping agents. Chitosan as a capping agent derived from different sources was reported to lead to a different degree of stabilization of Zn(OH)_2_ NPs against agglomeration and, hence, the crystal size. The crystallite size of ZnO NPs coated with chitosan derived from shells, crab shells, and *Streptomyces griseus* bacteria were observed to vary between 30.9 and 35.8 nm [27]. The crystallite size of ZnO/CS due to the surface coating with the biopolymer was observed to induce a reduction in the intensities of the peaks with crystallite size of 45 nm [21], similarly due to the broad stuff of chitosan at 22° in a study by Agnes [46] to possess the crystallite size of 21.45 nm and 16.57 nm for CS-ZnO and FA-CS-ZnO NPs, respectively.

The zeta potential (ζ-potential) of biosynthesized TM@ZnO/TMC NC hydrogel investigated using the DLS technique was estimated at 23.5 ± 5.13 mV (Figure 3b). The ζ-potential as a measurement was established to support the dispersion capacity and surface charges associated with as-synthesized NC. A low zeta potential value indicates the possibility of flocculated dispersion that could result in particle coagulation and aggregation. Similar positive zeta potentials of 19.1 ± 6.6 mV and 29.3 ± 5.56 mV were reported for chitosan–ZnO and folic-acid-coated chitosan–ZnO NPs, respectively [46]. Also, another study shows that the ZnO NPs of 50 nm size modified with chitosan have the highest ζ-potential of 21.7 ± 1.6 mV, as compared against the other smaller-sized ZnO NPs of 18 nm with 15.4 ± 0.8 mV and 10 nm ZnO of 11.1 ± 4.4 mV, thereby indicating the direct relation between particles size and surface charges [47]. Similarly, in our case, the TM@ZnO/TMC NC with 57 nm-sized particles has the highest ζ-potential of 23.5 mV.

The surface analysis of the TM-mediated ZnO-coated TMC determined from the Brunauer–Emmett–Teller (BET) technique, as shown in Figure 3c,d, indicated the creation of TM@ZnO/TMC NC hydrogel with a surface area of 3.01 m^2^ g^−1^, a pore volume of 0.6918 cm^3^ g^−1^, and an average pore size of 22.93 nm. In related study, ZnO NPs formed using the extracts of *Ziziphus Jujube* plant and assisted by ultrasonic irradiation gave a BET surface area of 14.23 m^2^ g^−1^, with average pore volume, and pore diameter of 0.000463 cm^3^ g^−1^ and 28.15 nm, respectively [25]. The isotherms of ZnO NPs were assigned as type II isotherm in the study by Tijani et al. [48], who reported the macroporous nature of ZnO with surface area of 8.99 m^2^ g^−1^, volume of 0.353 cm^3^ g^−1^, and pore diameter of 13.17 nm. A similar report consists of surface area for ZnO tetrapods prepared by the combustion method in the range 8−22 m^2^ g^−1^ [49]. In some studies, the surface coating and annealing temperature were reported to influence the surface area and pore size of ZnO NPs [42,50]. The surface analysis of as-synthesized mesoporous ZnO on varying the annealing temperature from 275 to 600 °C was observed to give the particles with surface areas of 25.3641 to 8.7781 m^2^ g^−1^ and mean diameter of 16.03 to 25.03 nm [50]. Similarly, varying pore sizes of 3.905, 4.024, 3.897, 3.745, and 3.626 nm were reported for various standards of chitosan samples, such as chitosan powder, its pure beads, the beads mixed ZnO, and ZnO NPs-coated beads, respectively. The decrease in the pore size recorded for the ZnO NPs-coated beads as compared to the other materials according to the study emanated from the increase in the surface area following the addition of ZnO particles. Further, this was reported to demonstrate varying surface areas of 9.7852, 1.7461, and 2.2436 m^2^ g^−1^ for ZnO NPs, chitosan, and ZnO/chitosan NC, respectively. The mean pore size of the ZnO/chitosan was reported as 12.2 nm, slightly higher by a factor than that of ZnO NPs (11.34 nm) and significantly lower than that of pristine chitosan (21.68 nm) [43].

### 2.3. Studies of Adsorption Kinetics

**Figure 4:**
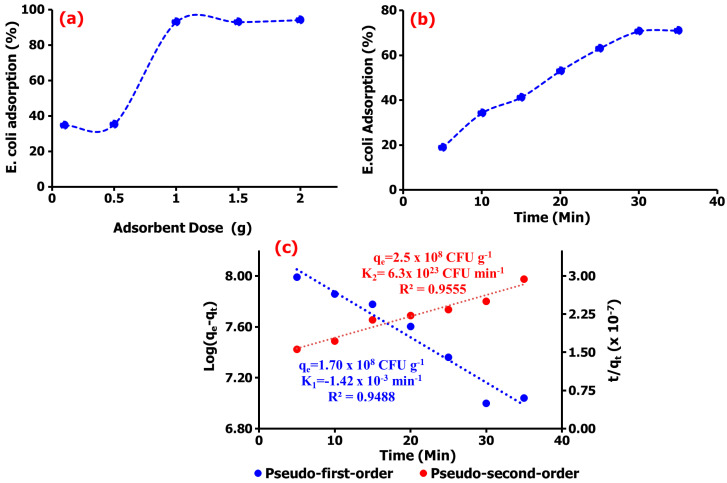
(**a**) Dose-dependent adsorption, (**b**) the percentage adsorption, and (**c**) the kinetics analysis of *E. coli* adsorption onto TM@ZnO/TMC NC hydrogel.

The adsorption study displayed in Figure 4a demonstrated to have a dose-dependent *E. coli* adsorption of ~70% onto TM@ZnO/TMC NC hydrogel in the aqueous suspension within a short interaction time of 5–35 min (Figure 4b). The sorption sites created by the addition of amine and carboxylate functional groups from the surface coating with TMC allowed for a systemic or gradual adsorption behavior, as shown in the figure. The presence of quaternary ammonium compounds on the surface of NPs were reported to aid the microfiltration of *E. coli* and observed to demonstrate 90% removal efficiency [51]. The adsorption kinetics established over the prescribed time was utilized in this work to investigate the sorption mechanism of *E. coli* onto TM@ZnO/TMC NC hydrogel. From the short interaction time, the kinetic study, as shown in Figure 4c, was observed to best follow the pseudo-second-order mechanism with rate constant and removal rate of 2.5 × 10^8^ CFU g^−1^ and 6.3 × 10^23^ CFU min^−1^, respectively. This demonstrated the highest level of linearity (0.9555) in comparison with the determination by the first-order adsorption model (0.9488), thereby predicting chemisorption as the rate-limiting step describing the adsorption processes [52], thus serving as the best kinetic model to describe the adsorption of *E. coli* onto TM@ZnO/TMC NC hydrogel, supporting the claim of pseudo-second order, describing the equilibrium of *E. coli* adsorption onto the surface of NC hydrogel [2,53].

### 2.4. Studies of Adsorption Isotherm

**Figure 5:**
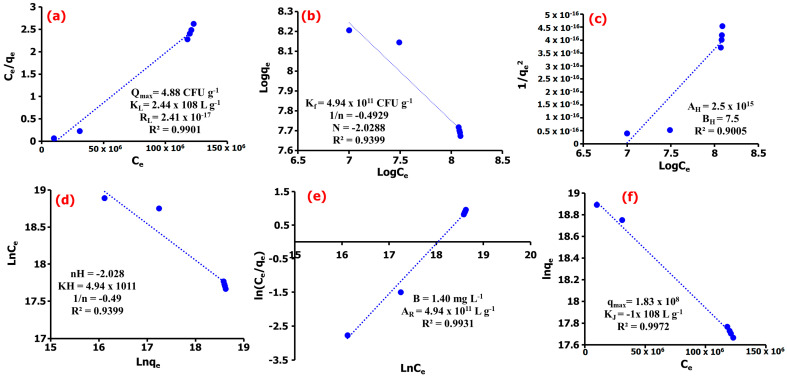
(**a**) Langmuir, (**b**) Freundlich, (**c**) Harkin–Jura, (**d**) Halsey, (**e**) Redlich–Peterson, (**f**) Jovanovic adsorption isotherm models of *E. coli* adsorption onto TM@ZnO/TMC NC hydrogel.

The primary adsorption mechanism was determined further using the Langmuir, Freundlich, Harkin–Jura, Halsey, Redlich–Peterson, and Jovanovic isotherm models. Comparing the obtained goodness-of-fit values for the Langmuir and Freundlich models, the experimental data as presented in Figure 5a,b show the adsorption of *E. coli* onto the NC surface well described by the Langmuir adsorption isotherm (R^2^ = 0.9901) compared to the Freundlich model (R^2^ = 0.9399), suggesting majorly the existence of a homogeneous adsorption processes [54]. Thus, the adsorption results generated from this study suggested that the adsorption process predominantly occurs homogeneously at the surface sites of TM@ZnO/TMC NC hydrogel and that the adsorption of *E. coli* may be assumed to be of monolayer coverage [42]. The experimental data generate a Langmuir constant (K_L_) of 2.44 × 10^8^ CFU g^−1^, which compliments the affinity of TM@ZnO/TMC NC hydrogel for *E. coli* with a maximum adsorption (q_max_) value of 4.90 CFU g^−1^. The calculated separation factor (R_L_) of 2.41 × 10^−17^ for *E. coli* adsorption on TM@ZnO/TMC NC hydrogel further suggests for the monolayer coverage process to be the dominating adsorption mechanism [55]. This finding followed similar other findings with the adsorption of *E. coli* onto NPs fitting well with the Langmuir isotherm model [53,56,57,58]. The lower values of R^2^ derived for Freundlich isotherm implied lesser applicability of adsorption onto a heterogeneous adsorbent surface and multilayered adsorption as, evidently described by the lower values of 1/n (−0.4929).

In order not to overrule the possibility of multilayer adsorption, the data were further subjected to Harkin–Jura and Halsey isotherm models, as shown in Figure 5c,d [59]. The R^2^ values determined for the adsorption isotherms were high but lesser than that of monomolecular adsorption models discussed earlier, revealing that the Hurkins–Jura model (R^2^ = 0.9005) weakly describes the experimental isotherm data in the sorption of *E. coli* onto TM@ZnO/TMC NC hydrogel. The Halsey model, which assumes a multilayer behavior for the sorption of sorbent onto the adsorbent, was also observed to show a lower fit for the adsorption of *E. coli* onto TM@ZnO/TMC NC hydrogel. This is revealing for the regression coefficient (R^2^= 0.9399) to be lower than those assumed for the monolayer adsorption process. Therefore, it can be concluded that *E. coli* adsorption onto TM@ZnO/TMC NC hydrogel is majorly driven by monolayer adsorption mechanism [41].

Further, the adsorption data has been analyzed by means of Redlich–Peterson and Jovanovic models so as to provide a satisfactory mechanism, where the results are shown in Figure 5e,f. This model provides a similar conclusion to the Langmuir model; however, the Redlich–Peterson model accommodates the determination of surface-binding vibrations of an adsorbed species [60]. The Redlich–Peterson serves as a bridge determined as a compromise for Langmuir and Freundlich adsorption processes to clarify the preeminence of either heterogeneous or homogenous adsorption in a system [61]. From the results provided in Figure 5e, the Redlich–Peterson constant (A_R_) of 4.94 × 10^11^ L g^−1^ and β values of 1.40 mg L^−1^ were determined for the adsorption of *E. coli*, suggesting that the TM@ZnO/TMC NC hydrogel as biosorbent has an affinity for *E. coli* in aqueous systems [60]. This assumed a compromised adsorption mechanism towards the Langmuir model, suggesting the predominance of a homogenous surface and monolayer adsorption of *E. coli* onto the TM@ZnO/TMC NC hydrogel. The Jovanovic model as an approximation was applied to describe adsorption mechanism with both mobile and localized monolayers without lateral interaction to show slightly higher R^2^ values (0.9972) as compared to the Redlich–Peterson and Langmuir models (Figure 5f). This model is also built upon the assumptions based on the Langmuir isotherm model [62]. The predominance of the surface homogeneity and monolayer adsorption nature of TM@ZnO/TMC NC hydrogel toward *E. coli* were also complemented by the higher q_max_ (1.83 × 10^8^ mg g^−1^) value determined [63]. Overall, the surface homogeneity and monolayer adsorption reflect well the entire adsorption mechanism, displaying the highest correlation for Langmuir’s and the Jovanovic and R–P isotherm models. The performance of TM@ZnO/TMC NC hydrogel based on the maximum adsorption capacity obtained from the Langmuir model was found comparable to, or in some cases better than, other adsorbents reported in the literature (Table 2). This is meant to support the utilization of the green route process without modification to appraise its efficacy as an adsorbent in future work against other bacterial strains.

### 2.5. E. coli–TM@ZnO/TMC NC Hydrogel Adsorption Interaction

Bacterial cell interactions with NPs surface occur through a combination of a balanced interparticle forces reported to be influenced by the material properties which include, but are not limited to, the surface charge heterogeneity, particle roughness, and the amount of surface coating agents. These material chemistries are also utilized in defining the NPs–bacterial surface interaction mechanism using various instruments [1]. However, for this study, the changes in the functional group bands before and after their interaction with *E. coli* investigated using the FTIR technique were used at least to draw out a preliminary information for the ZnO/TMC hydrogel involvement in the *E. coli* adsorption process. Thus, additional instrumental analysis is recommended to draw a clearer view of the NPs interaction mechanism with that of bacterial cells.

**Figure 6:**
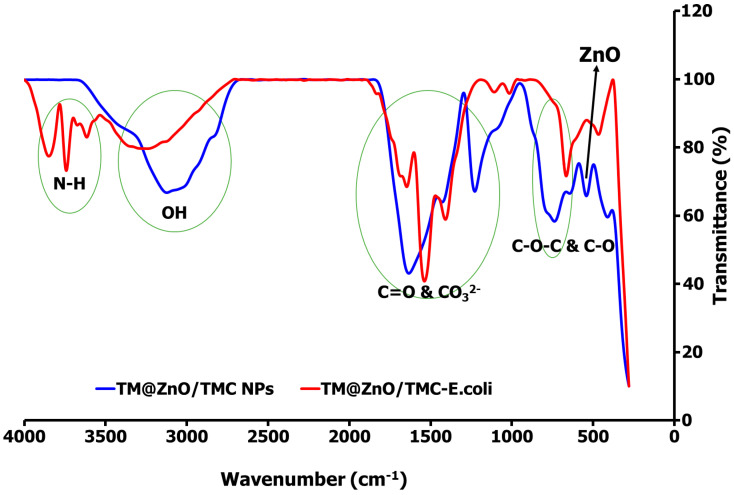
FTIR spectral comparison of TM@ZnO/TMC NC hydrogel before and after interaction with *E. coli*, demonstrating the adsorption mechanism.

From the R^2^ values determined for each adsorption model, the sorption of *E. coli* onto the TM@ZnO/TMC NC hydrogel is majorly driven by the formation of a monolayer structure, compatible with the mechanism described by the Langmuir, Jovanovic, and R–P models. The FTIR spectra shown in Figure 6 show the adsorption interactions of *E. coli* onto the TM@ZnO/TMC NC hydrogel. From the spectra, the successful interactions of TMC biopolymer onto the TM@ZnO NPs (TM@ZnO spectra shown in Figure 2) surface are assigned to the stretching vibrational activities of C–O and C–N of the amides (–NH_2_) at 1647 and 1234 cm^−1^ [45]. The band between 3000 and 3129 cm^−1^ in the spectra are identified to be from the amines and the hydroxyl groups stretching vibrations [43]. The presence of a band at 547 and 748 cm^−1^ in the spectra is a fingerprint of zinc-oxide vibration that underwent structural changes from the 471–614 cm^−1^ identified in the spectra of uncoated TM@ZnO NPs. Other authors also reported similar peaks indicating the surface coating of ZnO NPs with chitosan biopolymer [21,28,43,46].

The spectral profiles of TM@ZnO/TMC NC hydrogel on interaction with *E. coli* underwent some conformational changes in the broad peaks assigned to the stretching vibrations of the amines and –OH (3000–3129 cm ^−1^). In addition to the broad band of –OH peak centered at 3338 cm^−1^, the TM@ZnO/TMC NC hydrogel on interaction with *E. coli* produced additional peaks at 3619–3856 cm^−1^. These regions in the untreated bacterial profile are utilized as specific fingerprints in the identification of fecal *E. coli* activities using FTIR [68,69]. The peaks are associated with the stretching activities of N–H of amides in the protein structure of *E. coli*. The surface interactions of *E. coli* with NPs were reported to introduce spectral changes in the N-H, C-H, and the C-O-C and C-O stretching activities of the *E. coli* [68,69], further reported to introduce a spectral shift in the amide-protein region in bacteria-treated AgNPs [68] and *E. coli* exposed to quantum dot NPs [70]. In the spectra of TM@ZnO/TMC NC hydrogel displayed in Figure 6 is the peak at 1647 cm^−1^ following the interaction with *E. coli* increase by 6 cm^−1^ in wavelength to 1653 cm^−1^ in the spectra of TM@ZnO/TMC-*E. coli.* The vibrational activities in such a region were reported to be governed by the stretching effects of C=O of proteins sheet structures [68]. This is supported in another study to show C=O bonds at 1690 cm^−1^ in NPs-treated pathogenic bacteria [71]. Additional peaks at 1543–1416 cm^−1^ corresponding to CO_3_^2−^ stretching vibrations were also detected in the spectra of TM@ZnO/TMC-*E. coli*. Exposure of *E. coli* to NPs was reported to induce spectral changes from 1440 to 1460 cm^−1^ [68,72,73]. The peak at 1234 cm^−1^ in the spectra of TM@ZnO/TMC NC hydrogel was observed to split as 1000–1105 cm^−1^ in the spectra of TM@ZnO/TMC-*E. coli*. The bands at 1055 and 1058 cm^−1^ observed in the spectra of *E. coli* not exposed to NPs were reported to undergo a shift in wavelengths on exposure to Cu/Zn NPs, which identified the interactions to occur at the C-C bond of the carbohydrate region of the bacteria [68,74,75].

Similarly, the bands at 547 and 748 cm^−1^, a hallmark indication of Zn–O vibration in the spectra of TM@ZnO/TMC NC hydrogel, were observed to be shifted to a lower band at 671 cm^−1^ and 468 cm^−1^ which, according to some studies, reflects an interaction with the nucleic acids fingerprint of *E. coli* [68,76]. This interaction mechanism shown in the FTIR spectra, as reported in the foregoing literature, can weaken the *E. coli* cell membrane potential and integrity, enabling the binding of the NPs; further reported to impede cell wall hemostasis specifically by interacting with the bacteria sulfur-containing groups and in some cases elicited ROS formation from both OH and H_2_O_2_ and mismetallation of proteins in a cell [77,78,79].

## 3. Conclusions

The biosynthesis of TM@ZnO/TMC NC hydrogel provides a greener route for the production of a biosorbent for the adsorptive removal of *E. coli* in aqueous suspensions. The characterization of formed biosorbent confirmed the formation of NPs that are predominantly near-spherical in shape with a mean particle size of 31.81 ± 27.68 nm, BET surface area of 3.01 m^2^ g^−1^, and crystals size of 28.02 ± 7.72 nm that demonstrated to remove 70% of *E. coli* over a short time of 5–35 min, further observed to be preceded through a pseudo-second-order kinetic with rate constant and removal rate of 2.5 × 10^8^ CFU g^−1^ and 6.3 × 10^23^ CFU min^−1^, respectively. The Langmuir maximum adsorption capacity of 4.88 CFU g^−1^ and R_L_ of 2.41 × 10^−17^ indicated that the process was favorable and reflected the predominance of surface homogeneity and monolayer adsorption characteristics of the entire adsorption mechanism as complemented by the higher R^2^ values determined from the Jovanovic and the R–P isotherm models. From the results of this study, the TM@ZnO/TMC NC hydrogel as a low-cost and environmentally friendly biosorbent demonstrated the potential for *E. coli* removal from aqueous systems. However, for further sample application, the adsorption system will be examined in our future study using column technology under continuous flow system with the view to incorporating the cost-effectiveness of implementing this hydrogel for *E. coli* adsorption.

## 4. Materials and Methods

### 4.1. Collection and Preparation of Plant Extract

The TM leaves were collected within the campus of Adamawa State University Mubi, identified and verified by a Botanist in the Department of Botany, Adamawa State University Mubi. The thoroughly washed samples dried at 30 °C in clean ambience were made into powder using pestle and mortar. Furthermore, 10 g of the sample in 50 mL of water were allowed to boil under reflux conditions at 100 °C for 2 h and filtered through Whatmann No. 1 filter paper after allowing it to cool off for another 2 h. The aqueous filtrates collected were subsequently used for the synthesis of ZnO NPs.

### 4.2. Biosynthesis of TM@ZnO NPs 

The Zn metal precursor solution was prepared by dissolving 5.92 g of zinc nitrate hexahydrate (Zn(NO_3_)_2_⋅6H_2_O) in 200 mL of distilled water under stirring at about 1500 rpm for an hour at 60 °C. For the bioreduction process, varying concentrations of Zn(NO_3_)_2_⋅6H_2_O solution in 90 mL of distilled water were added dropwise to the 10 mL of TM aqueous leaves extract prepared earlier under constant stirring at 60 °C temperature for 2 h. At the completion of reaction, the formed dirty colored precipitate was allowed to settle for 24 h, and then centrifuged at 3000 rpm for 15 min. The formed precursor is then washed with deionized water repeatedly and oven dried at 60 °C and calcined at 350 °C for 2 h to obtain TM@ZnO NPs.

The parameters such as concentration of zinc salt and the pH of reaction are factored in the choice of synthesis route. The work by Thi Tran et al. [41] reported an onset of ZnO NPs formation at 0.001 M concentration of the zinc salt, progressively forming the precipitate at concentration equal to or larger than 0.01 M ZnO NPs. The synthesis using 0.1 M, 0.05 M, and 0.01 M concentrations of the Zn salts revealed an average diameter size in the order of 0.1 M > 0.05 M > 0.01 M respectively [44]. Thus for this study, the Zn(NO_3_)_2_⋅6H_2_O for the bio-reduction process was adopted at concentrations of 0.001 M, 0.01 M, 0.05 M, and 0.1 M. It was further reported that the ZnO NPs formation are also influenced by the reaction pH, i.e., a pH 5–7 and 8 were reported to support the formation of small NPs [41,44]. According to the study reported by Gherbi et al. [29], high pH (pH ≥ 9, 10) favors fast reduction rate and aggregation of NPs. Further analysis shows that the oxidation readily occurs at low pH ≤ 5, putting pH 6 and 7–8 as an ideal range for the formation of smaller sized NPs and hence for this study the reaction pH of 4, 6, 7, and 8 were adopted.

### 4.3. Synthesis of TM@ZnO/TMC NC Hydrogel

The formation of TM@ZnO/TMC NC was prepared based on modified procedures described by Bashal et al. [28], while the synthesis method by Mohammad et al. [45] was adopted for TMC and its hydrogel formation. For the formation of TMC, low molecular weight chitosan (4 g), sodium iodide (9.6 g), iodomethane (23 mL) and 15% of aqueous solution of sodium hydroxide (22 mL) were all thoroughly mixed in water bath in 160 mL of N-methyl-2-pyrrolidinone. The clear solution of the precursor was further subjected to heating for 1 h at 60 °C under reflux, allowed to cool off under ambient condition and separated by precipitation with ethanol and isolated by centrifugation. The TMC powder was obtained by washing the formed precipitate in diethyl ether, followed by ethanol. Further, to approximately 15 g of the dried TMC powder in 100 mL of distilled water, 1.5 g of tripoly phosphate (TPP) in 50 mL of distilled water were added drop wise under stirring for an hour and the product separated by centrifugation after the addition of ethanol, forming the TMC powder. In the formation of the TM@ZnO/TMC NC hydrogel, TMC (1% *w*/*v*, 0.1 g) powder dissolved in aqueous acetic acid (1% *v*/*v*, 10 mL) solution was prepared at room temperature under stirring followed by sonication for 30 min at 50 °C. To the solution, 20 (*w*/*v*%) of ZnO was added gradually under vigorous stirring for 2 h, centrifuged at 350 rpm for 30 min, and the resulting precipitate was rinsed with methanol and oven-dried at 50 °C for an hour to obtain TM@ZnO/TMC NC hydrogel in its powdered form [45].

### 4.4. Phytochemical Analysis of TM Aqueous Leaf Extracts

The aqueous extract of TM leaves was analyzed for the presence of phytoconstituents according to the described standard procedure [62]. The qualitative study using 50 mg/mL of the TM aqueous leaf extracts was established either through color changes, precipitation or frothing exerted by the plant’s metabolites, and the reactive species of the respective reagents [17]. Mayer’s reagent was used for the determination of alkaloids, Shinoda test method for flavonoids, and Fehling solution for the determination of glycosides. The froth test and Borntrager’s reaction test methods were used for the analysis of saponins and anthraquinones, while ferric chloride tests were used for the tannins and the phenols. Liebermann–Burchard test methods were used for the determination of terpenoids and steroids. Furthermore, about 2 g of the sample dissolved in a 1.0 mL vial containing hexane were used for the quantitative analysis. The phytonutrients were analyzed by injecting the said samples into a high-performance liquid chromatography (HPLC) system (bulk scientific BLC10/11, USA) with a fluorescence detector (excitation at 295 nm and emission at 325 nm) and analytical silica column (25 cm × 4.6 mm ID, stainless steel 5 µm). A ratio of 1000:60:4 *v*/*v*/*v* of hexane:tetrahydrofuran:isopropanol was used as the mobile phase, ran at a flow rate of 1.0 mL/min. Standard samples were also prepared using a similar method, and the concentration of phytonutrients in the sample was calibrated using some authentic standards.

### 4.5. Bacterial Adsorption Studies 

For the *E. coli* adsorption study onto ZnO/TMC NC, a modified protocol described in Bwatanglang et al. [2] was adopted. A known concentration of TM@ZnO/TMC NC hydrogel in a media containing cultured bacterial cell lines (*E. coli*) in nutrient broth (NB) was first incubated at 35 °C for 24 h at 100 rpm under continuous shaking conditions.

From this, approximately 1 mL of the bacterial/NC suspension was withdrawn at a predetermined time and the percentage removal efficiency of the *E. coli* (Initial concentration of 1.7 × 10^8^ CFU/mL (CFU: colony forming unit)) adsorbed onto the TM@ZnO/TMC were estimated from the relationship R%=[(Ci−Ce)/Ci]×100, where *Ci* and *Ce* are the initial and equilibrium concentrations of *E. coli* (CFU/mL). The uptake capacity (CFU/g) of the sorbents for each concentration of *E. coli* at equilibrium was determined from the equation qe (CFU/g)=[Ci−Ce)/M]×V, where *V* is the volume of the solution in L, while *M* is the mass of biosorbent (g). The kinetic and adsorption mechanisms were determined using the relationships described in Ojediran et al. [6] and Bwatanglang et al. [2]. The parameters are presented in Table 3.

### 4.6. Instrumental Analysis

The TMC uncoated TM@ZnO NPs and the TM@ZnO/TMC NC hydrogel were subjected to various stages of analysis using the following instruments. A Micro-meritics Tristar II Plus (Micromeritics, Norcross, GA, USA) BET machine was used for the surface analysis using N_2_ adsorption at 77 K. The FTIR spectroscopy using PerkinElmer (Waltham, MA, USA, N3895) was used for the functional group’s identification. The FESEM on JEOL JSM-7600F (JEOL, Tokyo, Japan) were employed for the determination of the particle shape and morphology, while the particles diameter in the solution phase was measured using DLS instruments, Malvern Nano series, Zetasizer instrument (Malvern Instruments, Malvern, UK). A XRD instrument (Malvern PA Analytical Empyrean, Almelo, The Nederlands) equipped with Cu anode (Cu Kα radiation source) X-ray tube was deployed for the phase identification and the determination of the structure. The quantitative phytochemical analysis was carried out using a HPLC (Bulk Scientific BLC10/11, Norwalk, CT, USA).

## Figures and Tables

**Table 1:** Results of qualitative and quantitative phytochemical analysis of TM aqueous leaf extract.

Qualitative analysis								
Tannins	Saponins	Flavonoid	Terpenoids	Alkaloids	Glycosides	Steroids	Phenols	Anthraquinones
++	+++	++	+	+++	++	+	+++	+
Quantitative analysis (mg/100 g)								
6.92 ± 0.04	6.54 ± 0.01	2.21 ± 0.02	1.34 ± 0.01	8.71 ± 0.02	3.28 ± 0.05	0.95 ± 0.02	36.55 ± 0.01	1.24 ± 0.01

Key: + slightly present, ++ present, +++ highly present.

**Table 2:** Comparison of the maximum sorption capacity (q_m_) of *E. coli* onto TM@ZnO/TMC NC hydrogel with those of other adsorbents.

Adsorbents	Adsorption Maximum (q_m_)	Reference
Silicon carbide	8.26 × 10^10^ mg g^−1^	[56]
Sulphate calcined ES	1.56 × 10^9^ mg g^−1^	[64]
Pt(IV) Binary solution	3.32 × 10 mg g^−1^	[58]
Pd (II) binary solution	7.32 × 10 mg g^−1^	
Fe_3_O_4_B	6.16 × 10^10^ mg g^−1^	[53]
Limestone	2.50 × 10^3^ mg g^−1^	[65]
Laterite soil	3.33 × 10^3^ mg g^−1^	
SiO_2_@NH_2_@COOHCST	5.20 × 10^9^ mg g^−1^	[66]
Single-wall carbon nanotubes	3.33 × 10^10^ mg g^−1^	[67]
ESCaCO_3_	9.54 × 10 mg g^−1^	[2]
RHSiO_2_	1.18 × 10 mg g^−1^	
CS−SiO_2_/CaCO_3_	3.18 × 10 mg g^−1^	
TM@ZnO/TMC NC hydrogel	4.90 × 10 mg g^−1^	This study

**Table 3:** Parameters for kinetic and adsorption isotherm models.

Models	Linier Equations	Parameters
Pseudo-first-order kinetics	$Log\;\left(qe-qt\right)=Logqe-\frac{K1t}{2.303},$	*K*_1_(CFU/g): Rate constant
		*q_e_* (CFU/g): Adsorption capacity at equilibrium
		*q_t_* (CFU/g): Adsorption capacity at time *t*
		Plot: *Log* (*q_e_* − *q_t_*) versus *t*
Pseudo-second-order kinetics	$\frac{t}{qt}=\frac{1}{{K_{2}qe}^{2}}+\frac{1}{qt}\;t$	*K*_1_(CFU/g): Rate constant
		*q_e_* (CFU/g): Adsorption capacity at equilibrium.
		Plot: *t*/*qt* versus *t*
Langmuir isotherm	$\frac{Ce}{qe}=\frac{1}{qmKe}+\frac{Ce}{qm}$ Separation factor, $RL=\frac{1}{\left[KL\;Ci+1\right]}$	*q_e_*: Amount adsorbed at equilibrium (mg/g).
		*C_e_*: Equilibrium concentration of the adsorbate,
		*q_m_*: Langmuir constant (Maximum adsorption capacity)
		*K_e_*: Langmuir constants (Binding energy of adsorption).
		*C_i_*: Initial concentration
		*K_L_*: Concentration of Langmuir.
		*R_L_*: indicating a favorable adsorption process. If *R_L_* = 0, the adsorption is irreversible, is favorable when 0 < *R_L_* < 1, linear when *R_L_* = 1, and unfavorable when *R_L_* > 1.
		Plot: *Ce*/*qe* versus *Ce*
Freundlich isotherm	$Logqe=LogKf+\frac{I}{n}LogCe$	*K_f_*: Adsorption capacity of the adsorbent.
		1/n: Adsorption intensity of the adsorbent. Indicating the surface heterogeneity and favorability of the adsorption process. For 0 < 1/n < 1, the isotherm is favorable. A value of 1/n above one is indicative of unfavorable adsorption isotherms.
		Plot: *Logq_e_* versus *LogC_e_*
Redlich–Peterson	$Qe=K_{RP}Ce/(1+A_{R}\;C_{e}^{\beta }$)	KRP (L/g) and AR (L/mg) are Redlich–Peterson adsorption and affinity constants respectively. The constant β ranges between 0 and 1 and its represent Redlich–Peterson exponent; if β = 1, the model reduces to the Langmuir equation, and if β = 0, then Freundlich equation.
		Plot: In(*C_e_*/*q_e_*) versus *lnC_e_*
Jovanovic	$lnQ_{e}=lnQ_{max\;}-K_{J}C_{e}$	KJ is Jovanovic isotherm constant (L/g)
		Plot: *lnq_e_* versus *C_e_*
Harkin–Jura	$LogC_{e}\;\mathrm{versus}\;\frac{1}{q_{e}^{2}}$	A = Harkin–Jura parameter and B = constant where the isotherm constants are B (intercept/slope; mg^2^ L^−1^) and A (1/slope; g^2^ L^−1^) derived from the plots of logCe versus 1/*q_e_*^2^
Halsey	$logQe=\left(\frac{1}{nH}\right)lnC_{e}$	K_H_ is Halsey isotherm constant; nH is the Halsey isotherm exponent. Plot: *logq_e_* versus *lnC_e_*

## Data Availability

Data can be available from the first author upon official request and on requirement.

## Abbreviations

ZnO, zinc oxide; Zn, zinc; NC, nanohydrogel; NPs, nanoparticles; TM, Terminalia mantaly; TMC, trimethyl chitosan; E. coli, Escherichia coli; XRD, X-ray diffraction; FESEM, field-emission scanning electron microscopy; FTIR, Fourier transform infrared; UV-vis, ultra violet-visible; h, hours; °C, centigrade; CFU, colony forming units; eV, electron volts.
